# Mitragynine Attenuates Withdrawal Syndrome in Morphine-Withdrawn Zebrafish

**DOI:** 10.1371/journal.pone.0028340

**Published:** 2011-12-21

**Authors:** Beng-Siang Khor, Mohd Fadzly Amar Jamil, Mohamad Ilham Adenan, Alexander Chong Shu-Chien

**Affiliations:** 1 Malaysian Institute of Pharmaceuticals and Nutraceuticals, Malaysian Ministry of Science, Technology and Innovation, Bukit Gambir, Penang, Malaysia; 2 Forest Research Institute Malaysia (FRIM), Kepong, Selangor, Malaysia; 3 School of Biological Sciences, Universiti Sains Malaysia, Minden, Penang, Malaysia; Medical College of Wisconsin, United States of America

## Abstract

A major obstacle in treating drug addiction is the severity of opiate withdrawal syndrome, which can lead to unwanted relapse. Mitragynine is the major alkaloid compound found in leaves of *Mitragyna speciosa*, a plant widely used by opiate addicts to mitigate the harshness of drug withdrawal. A series of experiments was conducted to investigate the effect of mitragynine on anxiety behavior, cortisol level and expression of stress pathway related genes in zebrafish undergoing morphine withdrawal phase. Adult zebrafish were subjected to two weeks chronic morphine exposure at 1.5 mg/L, followed by withdrawal for 24 hours prior to tests. Using the novel tank diving tests, we first showed that morphine-withdrawn zebrafish display anxiety-related swimming behaviors such as decreased exploratory behavior and increased erratic movement. Morphine withdrawal also elevated whole-body cortisol levels, which confirms the phenotypic stress-like behaviors. Exposing morphine-withdrawn fish to mitragynine however attenuates majority of the stress-related swimming behaviors and concomitantly lower whole-body cortisol level. Using real-time PCR gene expression analysis, we also showed that mitragynine reduces the mRNA expression of corticotropin releasing factor receptors and prodynorphin in zebrafish brain during morphine withdrawal phase, revealing for the first time a possible link between mitragynine's ability to attenuate anxiety during opiate withdrawal with the stress-related corticotropin pathway.

## Introduction

Drug addiction is a disorder encompassing compulsive drug use and the inability to control drug intake [1]. Repeated exposure to drugs eventually results in initiation of physiological homeostatic adaptations to counter the undesirable effects of the exposure [2]. Upon cessation of drug intake, either voluntary or involuntary, the body will rapidly metabolize and clear the drug. However, the body's homeostatic counter adaptations do not diminish as rapidly, resulting in a complex phenomenon known as drug withdrawal [3]. Clinical symptoms associated with withdrawal include anxiety, headache, seizures, aches, hallucinations or influenza-like syndromes. The desire to avoid and escape from the aversive symptoms of withdrawal can unwantedly lead to opiate seeking, resulting in relapse. Hence, a major goal in addiction treatment is to relieve the severity of opiate withdrawal symptoms. Administration of opioid agonists such as methadone and buprenorphine are the most commonly used treatment against opioid withdrawal [4]. However long term usage of these opiate-like substances might instigate another phase of undesirable opiate dependence and abuse [5]. Natural products are a potential source of novel and safer compounds useful for drug withdrawal management [6]. Elsewhere, there are documented cases of herbal-based medicines as effective remedy in drug addiction treatment [7], [8].

*Mitragyna speciosa* Kroth or ‘Kratom’, a native Southeast Asia plant, has a well-documented history as remedy against various ailments including diarrhea, cough, muscular pain and fatigue [9]. *M. speciosa* is also frequently used by drug addicts seeking for relief during opioid withdrawal stage [10]. While the use of *M. speciosa* is illegal in Thailand, Malaysia, South Korea and Australia, it is as source of unregulated ‘legal high’ in the UK and USA [11]. Among contributing factors are the lower cost of the plant material as compared to buprenorphine and the widespread availability of Kratom vendors [12]. At least 25 alkaloid compounds have been isolated from Kratom leaves [13]. Mitragynine, an indole alkaloid, is the foremost compound present in Kratom leaf extracts. Collectively, studies have shown that mitragynine possess the ability to suppress withdrawal syndrome, together with antinociceptive and antidepressant properties [14], [15], [16]. However, there is still a paucity of understanding on how mitragynine exerts these effects.

The zebrafish is gaining reputation as an advantageous model for elucidating the neurobiology of drug addiction and withdrawal [17]. Phenotypically, zebrafish show robust place preference behavior when exposed to morphine [18], [19]. The potential of zebrafish was further validated by evidence showing addiction-induced dopaminergic projections in the zebrafish forebrain analogous to the mammalian mesolimbic system [20]. Withdrawal from psychotropic drugs such as morphine, diazepam and caffeine also evoked a series of anxiogenic behaviors and stress-related endocrine response in zebrafish [17]. The influence of sex differences on withdrawal syndrome in zebrafish also parallels findings in rodents and human [21]. Other positive factors accompanying the use of zebrafish are the existence of useful tools such as genetic manipulations, useful mutant strains and amenability to the development of high-throughput screening assays.

In mammals, the corticotropin-releasing factor (CRF) is responsible for the activation of the hypothalamic-pituitary-adrenal (HPA) axis, which makes the CRF system a major regulator of endocrine and behavioral responses to stress [22]. Subsequent studies have since revealed the involvement of the CRF pathway in the development of anxiogenic traits during drug withdrawal phase [23]. Since anxiety is the most visible trait and potentially the major driving force in precipitating relapses, the ability to manipulate CRF signaling may yield therapeutic benefits. CRF signaling is mediated through the activities of two distinct G-protein coupled receptor subtypes, known as CRF-R1 and CRF-R2 [24], [25]. Indeed, studies have demonstrated the ability of CRF receptor antagonists in reducing negative motivational effects during withdrawal phase [26], [27]. The attenuation of aversive effects of opiate withdrawal in CRF-R1 and CRF-R2 deficient mice further validates the role of the CRF pathway in addiction and withdrawal [28], [29].

Dynorphin is a kappa-opioid receptor system (KOR) activating opioid peptide implicated in several stress-induced behavioral responses including anxiety, depression and drug seeking behaviors [30]. Studies in different animal models have also ascertained the role of dynorphin and the KOR pathways in reinstatement of drug seeking behaviors [31]. Several studies have collectively established the relationship between the CRF and dynorphin pathways by showing that activation of CRF receptors provokes the downstream release of dynorphin, leading to the dysphoria state commonly associated with drug withdrawal [32]. It is therefore plausible that stress-induced anxiety and reinstatement of drug seeking is predominantly mitigated through a CRF-induced KOR dependent pathway [33].

Using zebrafish as a model system, we conducted a series of experiments to gain further insights into the actions of mitragynine during morphine withdrawal phase. We showed that mitragynine was able to reduce anxiogenic swiming behaviors and concomitantly lower whole body cortisol in morphine-withdrawn zebrafish. Real-time gene expression analysis also revealed a reduction in the expression of CRF-R1, CRF-R2 and prodynorphin (PDYN) genes when morphine-withdrawn zebrafish were exposed to mitragynine.

## Materials and Methods

### Fish maintenance

Wild type zebrafish (*Danio rerio*) were maintained in 16 cm×10 cm×27 cm tanks in a stand alone automated zebrafish housing system (Techniplast, Italy). Water temperature was maintained at 27–28°C and tanks were located in a room with 12 h light/dark cycle. Fish were fed twice per day with live brine shrimp and commercial fish pellets (Sanyu, Malaysia). Breeding and embryo rearing were as described by Westerfield [34]. Test animals were obtained from in-house population. For all experiments, 4–5 months old adult male and female zebrafish (50–50) were used [17].

### Conditioned place preference (CPP) for morphine test

The CPP test was carried out to validate the preference behavior for morphine in zebrafish [18], [35]. Tests were conducted in an isolated room between 1000 h to1800 h. The CPP tank was sized at 20.5 cm×12.5 cm×11 cm and divided into 2 halves, consisting of a white half and a black-dotted half, respectively.

During day 1 of the CPP experiment, a baseline preference test was carried out to determine the initial preference of fish towards the preferred compartment of the CPP tank [35]. Test animals were fed in the usual manner in the morning, with behavioral observations conducted 2 h later. Test fish was first introduced into the middle section of the test tank, allowed to habituate for 10 min before a 5 min visual recording to determine its baseline preference for which half of the tank.

Conditioning of fish with morphine was carried out by exposing fish to morphine for 30 min in its respective non-preferred compartment [35]. Fish that showed a baseline preference for the white half was gently netted to the dotted morphine side and left for 30 min, followed by exposure to system water on the white side for another 30 min. Similarly fish that showed baseline initial preference on dotted side were treated as above in a reversed manner for the same duration of time. Fishes were then returned to holding tank for 24 h without any feeding, to avoid unwanted variable response [35]. Only individuals with baseline preference between 50.1 and 79.9% for either side were chosen for subsequent experiments. This was done by excluding those with >80% time spent on either side during the baseline preference tests.

On day 2, the animal was reintroduced in the CPP tank to determine its preference for the white or dotted side in a morphine free environment with a 5 min visual recording. Result was expressed as percentage of changes in preference, calculated as percentage of time in morphine side after conditioning minus the percentage of time in baseline preference. For control, the whole CPP procedure was same, except that the fishes were exposed to non-morphine water during conditioning.

Separate tanks and fish nets were used during all transferring process to prevent the cross-contamination [35]. A total of three morphine concentrations (0.5, 1.5 and 3 mg/L) were tested. Morphine solutions were prepared by dissolving a morphine stock solution (10 mg/L) with system water. All behaviors of the fish during test was recorded using a Noldus EthoVision XT 8 system (Noldus Information Technology, Netherlands) with the camera placed approximately 1.2 m above the test tanks.

### Morphine treatment, withdrawal inducement and mitragynine preparation

The following protocol was used for morphine addiction and withdrawal. A total of 10 adult fish (1.1±0.2 g) was randomly chosen from the holding tank and chronically treated in treatment tank containing 1.5 mg/L of morphine sulphate (Lipomed, Germany) for two weeks [36]. In order to induce morphine withdrawal, fish were treated with 1.5 mg/L morphine for 2 weeks and transferred to system water for 24 h before experiment [36]. For control group, fish were kept in system water for two weeks, followed by an additional 24 h. For mitragynine treatment, morphine withdrawn fish were exposed to mitragynine (1 mg/L and 2 mg/L) for 1 h before subjected to the system water prior to experiment. For acute mitragynine treatment, fish were treated with 1 mg/L and 2 mg/L of mitragynine for 20 min before initiation of behavior testing. Feeding was carried out as usual throughout the whole treatment duration. For all experiments, adult fish aged 4–5 months old and a 50∶50 mixture of male∶female fish were used.

Mitragynine was obtained from fresh leaves of *M. speciosa* sampled from Kedah and Perlis, Malaysia. Upon authentication, samples were deposited at Malaysian Institute of Pharmaceuticals and Nutraceuticals (specimen voucher code number IPHARM-49-35-C1). Leaves were washed and dried in oven at 40°C for 3 days, prior to grinding into powder form. A total of 300–400 g of the dry powder was Soxhlet extracted in methanol at 40°C for 28 h. The suspension was filtered and methanol was removed by rotary evaporator. The methanol extract was further dissolved in 10% (v/v) acetic acid solution, filtered and washed with hexane. The solution was then treated with 25% (v/v) ammonia solution to raise pH to 9, followed by extraction with dichloromethane. Extracts were evaporated with rotary evaporator to produce the alkaloid extract. Isolation of mitragynine from alkaloid extract was carried using analytical and preparative HPLC as described elsewhere [37].

### Novel tank diving tests

Novel tank diving tests were performed according to Cachat et al. [36]. After the procedures of morphine treatment or morphine withdrawal or mitragynine treatment of morphine-withdrawal as described above, fish were placed in a 16 cm×10 cm×27 cm tanks filled with system water. Location of tests and visual system were as described above. Behavior of each fish was recorded for 6 min and the following endpoints were assessed: time spent in top of the tank (s); latency to top (amount of time it takes the fish to cross into the upper half of the tank; average entry duration (total time spent at top divided by the number of entries), top and bottom ratio, number of freezing bouts and duration of freezing bouts. Freezing was defined as total absence of movement except for the gills and eyes, for 1 s or longer [36]. The novel diving tests were also conducted on fish exposed to acute treatment of mitragynine.

### Whole body cortisol assay

An assay developed to measure zebrafish whole body cortisol was used to measure cortisol in fish exposed to chronic morphine treatment, morphine withdrawal and exposure of mitragynine on morphine-withdrawn fish [17]. Fish were immediately frozen in liquid nitrogen upon end of chemical treatment and stored at −20°C. Whole body was homogenized in 500 µL of ice-cold 1× PBS and the homogenizer rinsed with another 500 µL of PBS. The resulting homogenate was transferred to a glass tube, cortisol was extracted with diethyl ether (Fisher Scientific, USA), twice, and reconstituted with 1 mL of PBS. Enzyme-linked immunosorbent assay (ELISA) approach was used to quantify the cortisol concentration, performed using a commercially available human salivary cortisol assay kit (IBL International GMBH, Hamburg Germany). Absorbance was measured with a EnVision-2104 Multilabel Reader (Perkin Elmer, USA) using the manufacturer's software. The cortisol concentration was determined from standard curve derived from absorbance values of standard cortisol concentration curve. Concentrations were calculated as relative concentration per gram of body weight for each fish.

### Real-time PCR for mRNA expression analysis of zebrafish CRF-R1, CRF-R2 and PDYN

Fish subjected to chronic morphine treatment, morphine withdrawal and exposure of mitragynine on morphine-withdrawn fish were sampled for expression analysis of zebrafish CRF-R1, CRF-R2 and PDYN genes using real-time PCR. Brain tissues were dissected from the liquid nitrogen frozen fish and kept at −80°C prior to RNA isolation. Total RNA was isolated using TRIzol® reagent (Invitrogen, CA, USA) according to manufacturer's protocol. The integrity and purity of the RNA was verified by gel electrophoresis and ratio of 260/280 absorbance reading. RNA samples were stored at −80°C for downstream applications.

Specific primers for CRF-R1, CRF-R2, PDYN and β-actin were designed according to zebrafish sequences in GenBank (Table 1). Changes in the gene expression were quantified using semi-quantitative real time PCR approach. Before performing the PCR, total RNA was treated with RNase-free DNase (Promega Madison, WI, USA) in order to remove the genomic DNA contamination. PCR amplification was performed by using iScript™ One-Step RT-PCR kit on a CFX96 ™ Real-time PCR detection system (Bio-Rad, USA). Gradient PCR was first carried out to determine the optimized annealing temperature for each set of primers. Efficiency test was carried out to evaluate the performance of the respective primers sets. Real-time PCR amplification was carried out in a total volume of 25 µL mixture containing 12.5 µL of 2× SYBR Green Reaction Mix, 0.75 µL (0.3 µM) of each primer, 0.5 µL of iScript Reverse Transcriptase, 9.5 µL of nuclease-free H_2_0 and 1 µL of RNA template (100 ng). The PCR cycle was as follows: cDNA synthesis at 50°C for 10 min, default cycling conditions were used for reverse transcriptase inactivation at 95°C for 5 min, followed by 40 cycles of denaturation at 95°C for 10 s and annealing at 63°C for 30 s. Melt curve analysis was carried out to determine the specificity of the PCR products. The expression level of genes was calculated by using CFX Manager ™ software (BioRad, USA), with normalization to housekeeping gene β-actin. Three independent experiments were carried out and each experiment performed in three analytical replicates.

**Table 1:** Primer sequences used for semi-quantitative real time PCR gene expression study of corticotropin-releasing factor type 1 receptor, corticotropin-releasing factor type 2 receptor and prodynorphin.

Gene	Primer sequences 5′-3′	GenBank Accession number
CRF-R1	F: GGTGGCTGAGGGTGATGAAG	XM691254
	R: AGGCTTTCCAGTTCCCCAAA	
CRF-R2	F: CACATGGGCACTGAAGAGCA	XM002667848
	R: TGAGGGCTCCAACAGACACA	
PDYN	F: TGAGCCAGGCAGATTGTTCA	AF057040
	R:TCTCCGTCTGCGCCTGTATT	
β-actin	F: GGATTCGCTGGAGATGATGC	NM001001831
	R: CGTGCTCGATGGGGTACTTC	

Each sequence is listed starting from the 5′-end and its corresponding sequence ID are provided. F, forward primer; R, reverse primer.

### Statistical analysis

All data were presented as mean ± SEM (standard error of mean) of three independent experiments. Statistical analysis was performed using the one way analysis of variance (ANOVA) and where applicable, ranked by Tukey's multiple comparison tests. Values of p<0.05 were considered significant.

### Ethics statement

All procedures involving animal handling in this study complied with the guidelines of the Animal Ethics Committee, Malaysian Institute of Pharmaceuticals and Nutraceuticals, Malaysian Ministry of Science, Technology and Innovation and was approved by the same commitee under the File ID of PA/ACSC/002/2011.

## Results

### Zebrafish exhibit CPP towards morphine

**Figure 1:**
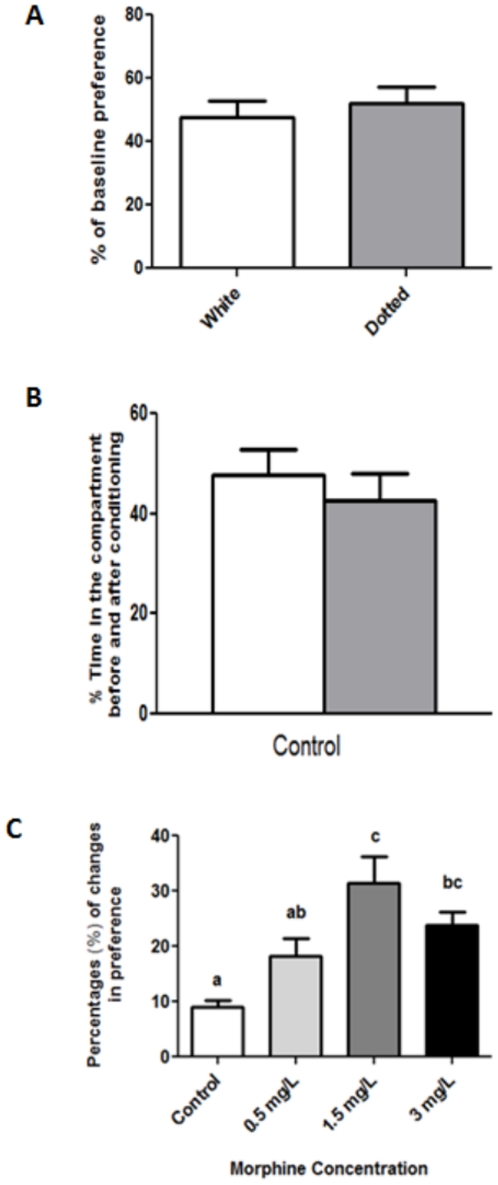
Conditioned Place Preference (CPP) test for morphine in adult zebrafish. (A) Baseline preference of fish between dotted vs. white compartment as shown by percentage of initial time spend in compartment (n = 23). (B) Preference behavior responses in the CPP tanks for control group as shown by percentage of time spent in conditioning compartment before (white bar) and after (grey bar) conditioning. (n = 15). (C) Place preference behavior of adult zebrafish treated different morphine concentration (Control, n = 15; 0.5 mg/L, n = 18; 1.5 mg/L, n = 16; 3 mg/L, n = 15). Mean values were subjected to one way ANOVA and where applicable, ranked by Tukey test (P<0.05); mean percentage values with same letter are not significantly different.

We first showed that zebrafish do not display significant preference for either dotted or white compartment (Figure 1A). In addition, control fish showed no differences in place preference before and after conditioning (Figure 1B). In comparison, morphine treatment at 0.5 mg/L and 1.5 mg/L enhanced the animal preference over an initially non-preferred compartment (Figure 1C). Reduced preference was obtained at the highest concentration (3 mg/L) (F_3,60_ = 8.109, P<0.001). This experiment validates the association of zebrafish with morphine as a positive stimulus at 1.5 mg/L, and this concentration was subsequently used in subsequent addiction and withdrawal experiments.

### Effects of morphine, morphine withdrawal and mitragynine on anxiety-related behavior in zebrafish

**Figure 2:**
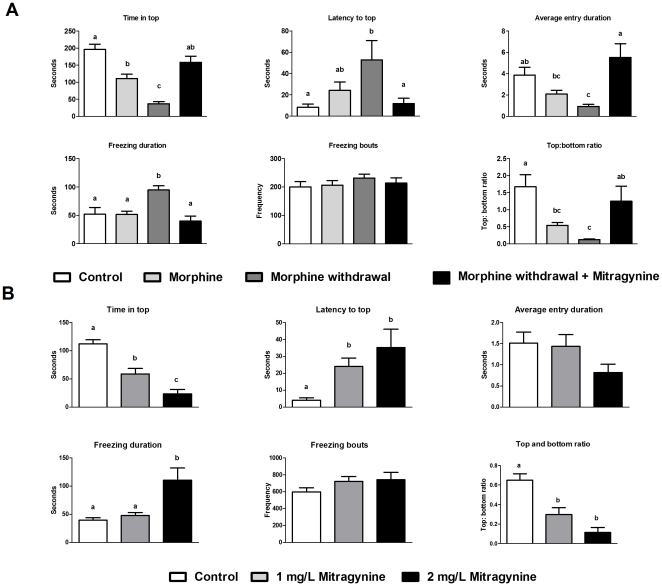
Effects of morphine treatment, morphine withdrawal and mitragynine on swimming behavior in adult zebrafish using the novel tank diving test. (A). Effect of two weeks chronic morphine treatment (1.5 mg/L), 24 h morphine withdrawal and morphine withdrawal in presence of 2 mg/L mitragynine on swimming behavior of zebrafish. (Control, n = 17; morphine treatments, n = 25; morphine withdrawal, n = 22; morphine withdrawal+mitragynine, n = 22). All data were presented in mean ± SEM. Mean values were subjected to one way ANOVA and where applicable, ranked by Tukey test (P<0.05); mean values with same letter are not significantly different. (B) Effect of 20 min acute mitragynine treatment (1 mg/L and 2 mg/L) on swimming behavior of zebrafish. (Control, n = 21; 1 mg/L mitragynine, n = 20; 2 mg/L mitragynine, n = 21). All data were presented in mean ± SEM. Mean values were subjected to one way ANOVA and where applicable, ranked by Tukey test (P<0.05); mean values with same letter are not significantly different.

Figure 2 shows the effect of two weeks of chronic morphine treatment, morphine withdrawal and mitragynine treatment of morphine-withdrawn fish on several behavioral indices of anxiety. Novel tank diving tests showed that there was a change in terms of the time spent at top and top∶bottom ratio between control and 2 weeks morphine treatment groups. No other apparent changes in other swimming behavior were observed. Overall, withdrawal from morphine produced strong anxiogenic behaviors in zebrafish. More specifically, morphine-withdrawn fish showed inferior explorative behavior as indicated by reduced amount of time spent at top of tank (F_3,82_ = 24.097, P<0.001), increased latency to reach the top of tank (F_3,82_ = 3.445, P = 0.020), reduced average entry duration (F_3,82_ = 7.243, P<0.001), and reduced top to bottom ratio (F_3,71_ = 5.931, P = 0.001). In addition, higher freezing duration (F_3,25_ = 9.026, P<0.01) and freezing bout values (F_3,82_ = 0.619, P = 0.605) also occurred in morphine-withdrawn fish. Collectively, these parameters indicate higher anxiety levels in morphine-withdrawn fish. In comparison, mitragynine treatment on morphine-withdrawn fish resulted in fish displaying swimming behaviors comparable to that of the control or morphine treated fish. There was no significant difference between control and mitragynine treated withdrawn fish for all the six swimming behaviors. Taken together, results from the novel tank diving tests imply that mitragynine attenuates stress-related behaviors in morphine-withdrawn zebrafish. Acute treatment of zebrafish with mitragynine, in particular at 2 mg/L resulted in changes in all the swimming behaviors (Figure 2B). For treatment with 1 mg/L, significant differences in terms of latency to top (F_2, 65_ = 5.243, P = 0.008), time in top (F_2, 59_ = 29.880, P<0.001) and top∶bottom ratio (F_2, 59_ = 18.912, P<0.001) as compared to control fishes were recorded, indicating a mild anxiolytic effect.

### Mitragynine lowers zebrafish whole-body cortisol production during morphine withdrawal phase

**Figure 3:**
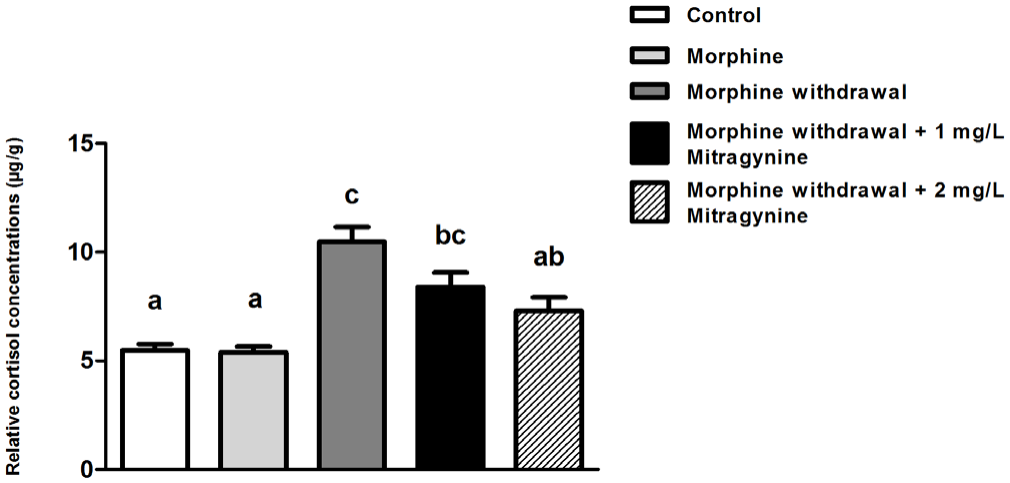
Effect of two weeks chronic morphine treatment (1.5 mg/L), 24-h morphine withdrawal and morphine withdrawal in presence of mitragynine (1 mg/L or 2 mg/L) on whole-body cortisol (µg/g fish). Data are presented as mean ± SEM. (Control, n = 16; morphine treatments, n = 14; morphine withdrawal, n = 21; 1.0 mg/L Mitragynine, n = 23; 2 mg/L Mitragynine, n = 20). Mean values were subjected to one way ANOVA and where applicable, ranked by Tukey test (P<0.05); values with same letter are not significantly different.

Significant elevation in cortisol level was detected in morphine-withdrawn zebrafish as compared to both control and chronic morphine treatment (Figure 3). However, exposing morphine-withdrawn fish to mitragynine at both concentrations significantly reduced whole-body cortisol level in zebrafish (F_4,89_ = 12.134, P<0.001).

### Changes in mRNA expression of CRFR and PDYN genes in morphine treated, morphine-withdrawal and mitragynine treatment of morphine-withdrawn fish

**Figure 4:**
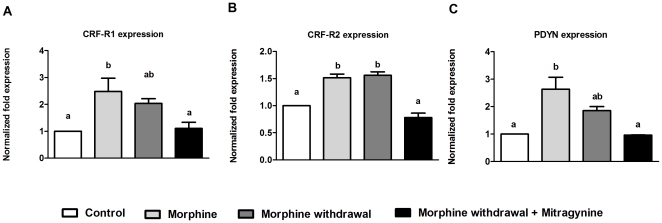
Effect of two weeks chronic morphine treatment (1.5 mg/L), 24-h morphine withdrawal and morphine withdrawal in presence of 2 mg/L mitragynine on the mRNA expression of CRF-R1, CRF-R2 and PDYN in zebrafish. Data are presented as mean ± SEM of three independent experiments. Mean values were subjected to one way ANOVA and where applicable, ranked by Tukey test (P<0.05); mean percentage values with same letter are not significantly different.

Transcripts of CRF-R1 increased significantly during chronic morphine treatment and reduced slightly during withdrawal phase (F_3,8_ = 6.373, P = 0.016) (Figure 4A). Treatment with mitragynine however reduced the expression to the level comparable with control fish. As for CRF-R2, significant high level of expression was obtained in morphine treated and morphine-withdrawn fish respectively while mitragynine treatment significantly lowered the level of CRF-R2 transcripts (F_3,12_ = 37.219, P<0.001) (Figure 4B). In general, PDYN showed a similar expression pattern with CRF-R1, with the highest expression in chronic treatment, followed by withdrawal and mitragynine treatment (F_3,4_ = 12.158, P = 0.018) (Figure 4C).

## Discussion

The harshness of opiate withdrawal symptom requires proper management strategies, which may include relieving stressful symptoms during withdrawal phase, to avoid relapse into addiction. Leaves of *M. speciosa* have been long used as a form of self-remedy among drug addicts to provide respite from withdrawal syndrome [10], [12]. Despite the extensive availability and use of this plant, much remains to be understood of *M. speciosa*'s ability to attenuate withdrawal symptoms [10], [13]. The zebrafish is a proven upcoming model for understanding drug withdrawal [38], [39]. Here, we investigate the influence of mitragynine, the main predominant alkaloid in *M. speciosa* on stress-related swimming behaviors, whole-body cortisol production and expression of CRF-R and PDYN genes in morphine-withdrawn zebrafish.

We first established the effect of morphine treatment on CPP behavior in adult zebrafish. Overall, our result recapitulates findings from earlier studies reporting the ability of morphine and other substances to enhance adult zebrafish's robust preference for an initially non-preferred compartment [18], [40]. The work of Lau et al. also showed increase in zebrafish brain morphine concentration after 15 min morphine exposure [18]. Using a series of novel tank diving tests, we then demonstrated that withdrawing zebrafish from morphine after a period of chronic exposure result in strong anxiogenic effects on zebrafish swimming behavior. These observations are consistent with a similar study reporting decreased exploratory behavior, reduced top transitions and increased erratic movement, freezing frequency and duration in zebrafish withdrawn from morphine [17]. Interestingly, the application of mitragynine significantly reduced the anxiety effects in morphine-withdrawn zebrafish, with several behaviors such as time in top of tank, latency to top and freezing duration being restored to levels before the onset of withdrawal treatment. These results parallel the known ability of *M. speciosa* in diminishing withdrawal symptoms [10]. Results from acute exposure of mitragynine alone seemed to indicate a mild change in swimming behavior effect. Elsewhere, it was reported that acute treatment of zebrafish with a range of *M. speciosa* leaf extract concentration resulted in mild sedative effect without any clear anxiolytic effect [39].

In teleosts, cortisol is the major product of physiological response to stress and is commonly used as a primary stress indicator [41], [42], [43]. Our results showed that while chronic treatment of morphine did not influence the cortisol level in the zebrafish, withdrawal from morphine significantly increases the cortisol level. Elsewhere, increased zebrafish whole-body cortisol secretion has been associated with withdrawal from several addictive substances, including ethanol and morphine [17]. In human, withdrawal from various addictive substances also elevates cortisol production [44], [45]. Furthermore, we showed that mitragynine significantly lowered whole-body cortisol production in morphine-withdrawn adult zebrafish, demonstrating an association between the attenuation of anxiogenic behaviors and lower production of cortisol in the presence of mitragynine. Administration of mitragynine also reduced corticosterone in mice, which gives an indication on the ability of this compound as antidepressant agent [15].

The release of cortisol is a result of a series of hormonal regulated events involving the activation of the hypothalamus-pituitary-interrenal axis, with the CRF acting as the initial hormone in the signaling cascade [46]. In zebrafish, the CRF is localized in different parts of the embryonic zebrafish brain, including telencephalon, preoptic region, hypothalamus, posterior tuberculum, thalamus, epiphysis, midbrain tegmentum, and rostral hindbrain [47]. In this present study, treatment with morphine increased the mRNA expression of both CRF-R1 and CRF-R2, which is consistent with observations in rats [26]. The expression of CRF-R1 was significantly lower in morphine-withdrawn fish compared to chronic morphine treatment, while no significant difference was observed for CRF-R2. This is reminiscent of the findings of Iredale et al. [26], where morphine-withdrawal reduced CRF-R1 expression in the rat brain. It has been speculated that the downregulation of CRF receptors during withdrawal is a homeostatic compensatory response against the overactivation of CRF-R1 during withdrawal, which adaptively lessens the arduous conditions of drug withdrawal. [26], [48], [49]. During chronic opiate exposure, an increase in cellular adenosine 3′,5′-monophosphate (cAMP) activities is a celluar measure to counter the inhibition of cAMP by opiates [50]. Therefore, the downregulation of CRF-R1 mRNA expression during morphine withdrawal is also a postulated homeostatic adaptation to counteract the overactivation of cAMP [51]. In addition, we also showed here that mitragynine significantly lowered the mRNA levels of both CRF-R1 and CRF-R2 in morphine-withdrawn fish, a trend that correlates with the attenuation of both anxiety behavior and whole cortisol production. The lower mRNA levels for both receptors in zebrafish treated with mitragynine is of importance, given the known fact that activation of both these receptors is shown to be partially responsible for stress-induced reinstatement of drug seeking [28], [52]. To our knowledge, this is the first study implicating a possible link between mitragynine and the CRF pathway in the mitigation of withdrawal symptoms, although the actual mechanism remains to be determined.

The zebrafish prodynorphin (PDYN) gene is highly homologous to the mammalian dynorphin and is capable of activating all known zebrafish opioid receptors [53]. In mice, both chronic morphine treatment and withdrawal increase dynorphin expression, which denote the importance of this protein in addiction and withdrawal [54]. In addition, the blockage of dynorphin with known antagonist decreased the severity of withdrawal [54]. Here, we showed chronic morphine treatment increased expression of zebrafish PDYN, while withdrawal from morphine and especially treatment with mitragynine significantly decreased the transcript levels. The reduced levels of zebrafish PDYN mRNA in presence of mitragynine could possibly explain the reduced stress-liked swimming behaviors displayed in the novel tank diving test as dynorphin, alongside CRF, is also a key mediator of stress-induced drug relapse during withdrawal [31]. In addition, the release of dynorphin by the activation of CRF receptors have been implicated as the cause of dysphoria state commonly associated with drug withdrawal [32]. Taking this into consideration, the lower expression of PDYN in zebrafish exposed to mitragynine could probably be the result of reduced expression of both the CRF receptors. In addition, it is worth mentioning that mitragynine possess affinity towards kappa-opioid receptors, which may hypothetically be an avenue for attenuation of negative withdrawal effects [10], [55].

In conclusion, we showed here the capacity of mitragynine to diminish stressful swimming behaviors and cortisol production in morphine-withdrawn zebrafish. In addition, using gene expression studies, we also showed for the first time that the attenuation of withdrawal syndrome by mitragynine might involve both the HPA and KOR pathways.
